# Nursing students’ satisfaction about their field of study

**Published:** 2014-04

**Authors:** ASHRAFALSADAT HAKIM

**Affiliations:** Chronic Disease Care Research Center, Nursing Department, Ahvaz Jundishapur University of Medical Sciences, Ahvaz, Iran

**Keywords:** Satisfaction, Nursing, Student

## Abstract

**Introduction**: Nowadays students' opinion is considered as a necessary factor to evaluate quality in universities. This study was performed to evaluate the nursing students' satisfaction about their field of study.

**Methods**: The research population in this study consists of all the students of nursing studying at the second to fourth year of university (72 students). The data were collected from all the studied population. Data collection instrument was a research questionnaire. In this cross-sectional research, nursing students' satisfaction (72 students) in 6 major topics (situation of educational environment, situation of clinical environment, trainers, social image, relation to colleagues and management) was studied. The data were analyzed in SPSS, version 14, using quantitative variables and descriptive statistics including frequency distribution tables and diagrams.

**Results**: The findings indicated that 83.3% of the students had little satisfaction as to the situation of educational environment, 47.2% about situation of clinical environment, 41.7% concerning the theoretical educational method by professors, and 41.7% as to the method of clinical education by clinical trainers. Also 47.2% were not that satisfied with the method of evaluation by the school professors, 80.6% with the method of relationship with colleagues and also 62.5% with the nursing social image. Moreover, findings indicated that 33.3% of the participants in this research were dissatisfied with the method of evaluation by clinical trainers and 50% with the method of nursing management.

**Conclusion**: In the present study, most students had little satisfaction concerning their field of study. So it is necessary to make an attempt for continuous development of quality services.

## Introduction

Satisfaction is the response of the customer to organizational success. In fact, satisfaction represents the level of customer’s pleasure in responding to the specificity of goods and services provided by the organization. Thus customer satisfaction is a major criterion in determining the quality provided through the process of services or product; also, it is a source of taking competitive advantage (1). In a competitive environment in which organizations compete with each other to attract customers, their satisfaction is a key element to succeed and an important objective of each organization is to achieve success because it leads to profitability and customers’ trust to the organization (2, 3). But in the case of dissatisfaction, this experience acts as a negative propaganda against the organization (4).

Therefore, evaluating satisfaction concerning the field of study in each college is considered among fundamental subjects of the college and a significant part of its activities in the area of organizational procedure and behavior. On the other hand, since interest in and satisfaction about the field of study is one of the most important factors in the students’ education and success, this issue should be in the top priority of educational planning (5). In this regard, also the field of nursing needs interested individuals with special abilities. Several studies have shown that the probation rate of students is more than their satisfaction of their filed (6). There is a correlation between the rate of satisfaction about the field of study and academic achievement in students (7). Given the very high occupational sensitivity of the medical sciences groups, and due to their direct relationship with human life and health of the community, the importance of their empowerment is double and any factor that can increase this capability is important (8). On the other hand, the studies have shown that 90 percent of students are satisfied with their field of study and have a positive view about it (9, 10).

It seems that some of these differences are relevant to the universities’ level and educational environment. Among other causes, these differences can be attributed to the universities’ educational programs and the role of schools, educational groups, and professors on improving attitude towards the field of study and the feeling of satisfaction about it (8).

One of the most important factors that could increase the students’ interest and motivation about their academic field of study  and also the graduates’ job satisfaction is the rate of satisfaction with the title of the field of study and the relevant job, social position, income and the difficulty of working in this field and job (11).

The results of a research performed in a medical sciences university indicate that 63.6% of students decided to change their field of study and 51.64% of them decided to choose resignation. Lack of positive social position is considered among dissatisfaction factors in nurses that can lead to frustration and resignation among students and avoidance of continuing the profession (12). Therefore, there are various factors that can create satisfaction about educational field in students and lack of one factor in this collection can decrease their satisfaction or even put them in a group of students who are dissatisfied about their field of study.

Numerous studies indicated that previous information of students about field of study, social image, duties description, job future, kind of relationship between university trainers, professors and hospital personnel with students, university type, college educational facilities, and related management methods are among the most important factors that lead to satisfaction about the educational field among students. According to the results of some research, there is a positive relationship between management processes and customer satisfaction. Moreover, the quality of duties performed has an important role in customer satisfaction. Customer satisfaction depends on consumers' experience and their reaction to the behavior of those presenting services in this process (13, 14). In another research, about 50% of the students were concerned about their future job and 35.5% of them had a negative viewpoint about their selected field of study (15). Therefore, the above mentioned issues prompted the researcher to perform a study with the aim of evaluating nursing students' satisfaction about the field of study in Nursing and Midwifery School.

The results of this study will hopefully increase the managers’ awareness and information about present shortcomings in educational system, qualitative and quantitative development of services, qualitative development of observations, improvement of the society’s health level and increase in the students' satisfaction.

## Methods

This is a cross-sectional research performed in one of Nursing and Midwifery Schools in Ahvaz Jundishapur University of Medical Sciences in 2012. In this research, nursing students' satisfaction was studied (72 students) in 6 major topics (situation of educational environment, situation of clinical environment, trainers, social image, relation to colleagues and management). The inclusion criteria in this research were the students studying at second to fourth years of school, consent to participate in the study, and filling out the questionnaire. The exclusion criteria included unwillingness to participate in the study or absence due to educational leave at the time of the study. This manuscript was approved by ethics committee of Jundishapur University of Medical Sciences. 

  The research population in this study consists of all the students of nursing studying at the second to fourth year of university (72 students). The data were collected from all the studied population. The data collection instrument was a research questionnaire, including 13 questions related to demographic features (education place, education situation, age, total average, dwelling, marital situation, father job, mother job), and 74 questions related to nursing students’ satisfaction level about their educational field and related factors in 6 major topics [situation of educational environment (question 1-30), situation of clinical environment (Question 31-46), trainers (question 47-51), social image (question 52-58), relation to colleagues (question 59-68) and nursing management (question 69-74)] were analyzed. For each question, answers were considered as completely dissatisfied, very little satisfaction, little satisfaction, average satisfaction, high satisfaction, very high satisfaction, and the data were collected by  after the agreement of the related organization with researcher presence in Nursing and Midwifery School (with necessary education about the method of filling out the questionnaire).

The method of scoring the answers was as follows: Score zero was allocated to answers of completely dissatisfied, score one to answers of very little satisfaction and scores two to answers of little satisfaction, score three to answers of average satisfaction, score four to answers of high satisfaction, and score five was given to answers of very high satisfaction. The minimum and the maximum scores of general satisfaction about educational field for each person were zero and 370; in different topics it was as follows: Situation of educational environment (School): from scores zero to 25 completely dissatisfied, scores 26 to 50 for very little satisfaction, scores 51 to 75 for little satisfaction, scores 76 to 100 for average satisfaction, scores 101 to 125 for high satisfaction, and scores 126 to 130 for very high satisfaction. 

Situation of clinical environment: from scores zero to 13 completely dissatisfied, scores 14 to 26 for very little satisfaction, scores 27 to 39 for little satisfaction, scores 40 to 52 for average satisfaction, scores 53 to 65 for high satisfaction, and scores 66 to 80 for very high satisfaction. 

Trainers: from zero to 4 for completely dissatisfied, 5 to 8 for very little satisfaction, 9 to 12 for little satisfaction, scores 13 to 16 for average satisfaction, scores 17 to 20 for high satisfaction, and scores 21 to 25 for very high satisfaction. 

Social image: from scores zero to 5 completely dissatisfied, scores 6 to 11 for very little satisfaction, scores 12 to 17 for little satisfaction, scores 18 to 23 for average satisfaction, scores 24 to 29 for high satisfaction, and scores 30 to 35 for very high satisfaction. 

Relation to colleagues: from scores zero to 8 completely dissatisfied, scores 9 to 16 for very little satisfaction, scores 17 to 24 for little satisfaction, scores 25 to 32 for average satisfaction, scores 33 to 40 for high satisfaction, and scores 41 to 50 for very high satisfaction. 

Nursing management: from scores zero to 5 for completely dissatisfied, scores 6 to 10 for very little satisfaction, scores 11 to 15 for little satisfaction, scores 16 to 20 for average satisfaction, scores 21 to 25 for high satisfaction, and scores 26 to 30 for very high satisfaction. 

In order to determine validity of the questionnaire, it was checked by several members of scientific board in Nursing and Midwifery School and their opinions were included. The questionnaire’s reliability was evaluated using the results of an elementary study on 25 students and for all items of questionnaire by using Cronbach's alpha coefficient, which was proved to be 94%. Moreover, this level was 91% for situation of educational environment, 71% for situation of clinical environment, 83% for trainers, 85% for social image, 88% for relation to colleagues and 86% for management, indicating an acceptable internal stability. 

In order to analyze the data, SPSS 14 (SPSS Inc, Chicago, IL, USA), was used for quantitative variables; also descriptive statistics including frequency distribution tables and diagrams were applied.

## Results

The percentages of students in the second, third and fourth years were 38.9 %, 38.9% and 22.2%, respectively. The students' mean age and also the mean grade point average (GPA) were 21.11±1.02 and 16.59±1.04, respectively. The percentages of students who lived in dormitory and were single were 97.2% and100 %, respectively. The percentage of the self-employed and housewife fathers and mothers as was 38.9% and 83.33%, respectively (Table1).

**Table 1:** Field of study and nursing students’ satisfaction according to demographic features

**Variable**	**Students (n=72)**
**Education year, n (%)**	
Second year	28 (38.9)
Third year	28 (38.9)
Fourth year	16 (22.2)
**Age (Mean±SD)**	21.11±1.02
**Grade point average of students' scores (Mean±SD)**	16.59±1.04
**Dwelling, n (%)**	
Dormitory	70 (97.2)
Non-dormitory	2 (2.8)
**Marital situation, n (%)**	
Single	72 (100.0)
Married	0
**Father's job, n (%)**	
Employee	20 (27.8)
Worker	6 (8.3)
Retired	16 (22.2)
Self-employed	28 (38.9)
Other	2 (2.8)
**Mother's job, n (%)**	
Employee	10 (13.90)
Housewives	60 (83.33)
Retired	0
self-employed	0
Other	2 (2.8)

The findings indicated that 83.3% of the students had little satisfaction concerning the situation of educational environment (School), 47.2% about situation of clinical environment, 41.7% as to the theoretical educational method by professors, and 41.7% as to the method of clinical education by clinical trainers. Also 47.2% were not that satisfied with the method of evaluation by the school professors, 80.6% with the method of relation with colleagues and also 62.5% with the nursing social image. Moreover, findings indicated that 33.3% of the participants in this research were dissatisfied with the method of evaluation by clinical trainers and 50% with the method of nursing management (Table 2).

**Table 2:** Field of study and nursing students’ satisfaction

**Variable**	**Students(n=72)**
**Situation of educational environment (School), n (%) **	
completely dissatisfied	0
very little satisfaction	12(16.7)
little satisfaction	60(83.3)
average satisfaction	0
high satisfaction	0
very high satisfaction	0
**Situation of clinical environment, n (%)**	
completely dissatisfied	12(16.7)
very little satisfaction	26(36.1)
little satisfaction	34(47.2)
average satisfaction	0
high satisfaction	0
very high satisfaction	0
**Theoretical education by School professor's, n (%) **	
completely dissatisfied	8(11.1)
very little satisfaction	10(13.9)
little satisfaction	30(41.7)
average satisfaction	11(15.3)
high satisfaction	13(18.1)
very high satisfaction	0
**Clinical education by clinical trainers, n (%)**	
completely dissatisfied	10(13.9)
very little satisfaction	19(26.4)
little satisfaction	30(41.7)
average satisfaction	13(18.1)
high satisfaction	0
very high satisfaction	0
**Evaluation by School professor's, n (%)**	
completely dissatisfied	12(16.7)
very little satisfaction	21(29.2)
little satisfaction	34(47.2)
average satisfaction	5(6.9)
high satisfaction	0
very high satisfaction	0
**Evaluation by clinical trainer's, n (%)**	
completely dissatisfied	24(33.3)
very little satisfaction	18(25.0)
little satisfaction	13(18.1)
average satisfaction	17(23.6)
high satisfaction	0
very high satisfaction	0
**Relation with colleague's, n (%)**	
completely dissatisfied	6(8.3)
very little satisfaction	7(9.7)
little satisfaction	58(80.6)
average satisfaction	1(1.4)
high satisfaction	0
very high satisfaction	0
**Social image, n (%)**	
completely dissatisfied	4(5.6)
very little satisfaction	20(27.8)
little satisfaction	45(62.5)
average satisfaction	3(4.2)
high satisfaction	0
very high satisfaction	0
**Nursing management n (%)**	
completely dissatisfied	36(50)
very little satisfaction	36(50)
little satisfaction	0
average satisfaction	0
high satisfaction	0
very high satisfaction	0

## Discussion

University students, as one of the major foundations of university, constitute the main structure of different organizations and systems in the community in future. The satisfaction of all performed activities in the university can be effective in their viewpoints about their educational field in order to create motivation and promote educational quality (16). In order to achieve this goal, this research was performed in Nursing and Midwifery School to evaluate the nursing students’ satisfaction from their field of study.

In general, in the present study, most students had little satisfaction concerning their field of study. While, according to the research findings about customer satisfaction, customer-based mean was 49.11 and minimum mean could not be obtained from 60 customers. It can be concluded that there is an equal proportion between satisfied and dissatisfied students in this university. So, 50% of the students in this university had satisfaction and 50% of them had no satisfaction; this accords with the findings of the present research (17).

The results also showed that satisfaction from the field of study is not at a desirable level (8). But in other studies the majority of students were interested in their field of study; this is inconsistent with the results of the present study (5). However, other studies indicated that more than 90 percent of students were satisfied about their field of study; they had positive attitude toward their field of study (9, 10).

Most of the students had little satisfaction about the situation of educational environment. In this regard, the results of a study on satisfaction level in the students of Saddleback College are as follows: education services, university present possibilities (66%), security of university environment (70%), and references existing in library (72%) (18). The results of this study are not in the same line with those of the present research; perhaps the reason of this difference is different research environments.

According to the research findings, most of In the study performed by Julae et al Inappropriateness of clinical work environment have been of priorities the reasons of leaving the field of nursing study (12). The best places to acquire this skill are university educational hospitals (19). Acquisition of basic and professional skills in medicine depends on the quality and quality of education in clinical environments (20). In a research, the shortage of competent trainers and use of trainers regardless of their abilities and expertise have been mentioned as clinical educational problems (21).

According to the findings of the present research, the majority of students had little satisfaction concerning the theoretical educational method used by professors. The results of the research performed about the students' satisfaction in the university of Liverpool in 2006 show the students viewpoints; the most and the least important factors were education, learning and physical facilities respectively and the level of satisfaction concerning the important sections was less than sections with the lower importance which the results of the research is in the same line with those of the present research (22). Moreover, in the study, the students showed no satisfaction about the educational program and the trainers' performance; this accords with the results of the present research (23).

The results of the present research indicated that most students had little satisfaction concerning the method of clinical education by trainers. The experts believe that clinical trainers have considerable effect on the increase of the quality of clinical education and they can make enjoyable clinical experiences for students. Therefore, it is necessary to further investigate the factors effective on developing motivation of clinical professors to find the reasons of their active and more effective attendance in educational and therapeutic field so that, the clinical professors use their valuable experiences to actively engage in clinical education (24).

Moreover, about causes, obstacles and problems of clinical education in nursing from the nursing students’ viewpoint on the performance of clinical instructors, the mean of students’ viewpoints was 17.72 from maximum score of 44 (25). In this way, other studies indicate that abilities and clinical skill of novice nurses to fulfill the needs and expectations of patients and health team and managers are insufficient (26). The present study findings showed that most of the nursing students were dissatisfied about the method of evaluation used by clinical trainers. While 52.75% of nursing and midwifery students evaluated the overall performance of clinical trainers at good and average levels, the results of this study are different; perhaps the cause of this disparity is the differences in educational environments (27).

The results of the research indicated that most of the students had little satisfaction concerning the method of relationship with colleagues, In the research, the survey of the students’ views in the topic of personnel relationship with students has been indicative of dissatisfaction. Lack of student support by the personnel, spleenful, nervous mood of the personnel, and lack of proper feedback by them to students are considered as the inhibiting factors. This can lead to the students’ apathy toward learning and creating negative attitude (28-30).

According to the results of the present research, most of the students were satisfied about the nursing social image. In many of the studies, negative attitudes of nurses and other health team personnel and strict routines of hospital wards are other factors reported for the leave of studying in nursing filed (30).

Also, Julae (2006) in his study has mentioned some of the main reasons behind leaving the field of study: lack of suitable social base, lack of coordination between facts and primary thoughts, lack of knowledge and information of people and society concerning this profession, and inappropriate work environment and professional areas (12). According to the findings of this research, half of the students were dissatisfied with the method of nursing management.

Since management in the level of educational centers especially in university level, from college up to ministry, has a vital and sensitive role to develop and achieve to the universities educational goals. In this regard, the researchers stated that in order to make constructive changes, the existence of descriptive information about the present situation and knowledge and awareness concerning students' opinion about the services delivered is necessary, so that using this information more student satisfaction (31). Thus for achieving the student satisfaction as customers or recipients of educational services, there should be an attempt to continuously improve the quality of services (32). In this regard, nursing researchers have also stated that the most important output of nursing science centers is providing the personnel with highest quality clinical services for clients. Therefore to achieve this goal, the mission of the nursing schools is empowering nursing students to accept important roles of nursing profession (33).

Given that these students are the future of nursing in our country, so it is necessary to consider the concerns and preoccupations of the nurses and their satisfaction. As such, these students will be able to continue their educational process with motivation and high quality; also, they will be able to readily attend the health care settings in the health services system. 

Students' emotional and psychological problems can be considered as one of the limitations of this study; it was actually outside the researcher's control. A similar research is suggested to be done in order to evaluate the satisfaction rate of other students in different medical sciences and then compare the results with the findings of this study.

## Conclusion

Since the students in the present study had little satisfaction about most topics and very little satisfaction concerning management topic, to achieve more students’ satisfaction in educational institutions, educational managers are recommended to make an attempt in the provision of continuous promotion of quality service.
